# *CHOP *5'UTR-c.279T>C and +nt30C>T variants are not associated with overweight condition or with tumors/cancer in Italians – a case-control study

**DOI:** 10.1186/1756-9966-28-90

**Published:** 2009-06-26

**Authors:** Ramachandran Meenakshisundaram, Nunzia Piumelli, Laura Pierpaoli, Claudia Gragnoli

**Affiliations:** 1 Department of Medicine and Cellular & Molecular Physiology and Biostatistics, Penn State Milton S. Hershey Medical Center, Hershey, Pennsylvania, USA; 2 Penn State University College of Medicine, Hershey, Pennsylvania, USA; 3 Sbarro Institute for Cancer Research and Molecular Medicine, Center for Biotechnology, Philadelphia, Pennsylvania, USA; 4 Department of Biology, Temple University's College of Science & Technology, Philadelphia, PA, USA; 5 Molecular Biology Laboratory, Bios Biotech Multi-Diagnostic Health Center, Rome, Italy

## Abstract

**Background:**

Type 2 diabetes (T2D) is associated with obesity and has been shown recently to be associated with tumors/cancer. *HNF1-beta *and *JAZF1 *genes are associated with T2D and prostate cancer. We have previously shown that *CHOP *5'UTR-c.279T>C and +nt30C>T haplotype variants contribute to T2D. CHOP deficiency causes obesity in mice, thus *CHOP *gene variants may contribute to human obesity. Furthermore, *CHOP *mediates apoptosis and is implicated in cancer pathogenesis. Hence, we aimed at identifying any potential association of *CHOP *5'UTR-c.279T>C and +nt30C>T genotypes and corresponding haplotypes with overweight condition/pre-obesity and tumors/cancer in an Italian dataset.

**Methods:**

We recruited from Italy 45 overweight subjects (body mass index (BMI) ≥ 25) and 44 control subjects (BMI < 25) as well as 54 cases with at least one cancer or at least one tumor and 43 control subjects without tumors/cancer from the general population. We excluded allelic departure from Hardy-Weinberg equilibrium in cases and control subjects, separately.

**Results:**

We assessed the power to detect risk odds ratios by association tests in our datasets. We tested the hypothesis of association of CHOP 5'UTR-c.279T>C and +nt30C>T genotypes and haplotypes with tumors/cancer and, separately, with overweight condition. Both associations were not significant.

**Conclusion:**

From our study, we may conclude that *CHOP *5'UTR-c.279T>C and +nt30C>T genotypes and corresponding haplotypes are not associated with tumors/cancer and pre-obesity. However, more studies are warranted to establish the role of *CHOP *variants in tumor/cancer predisposition and in overweight condition.

## Background

Type 2 diabetes (T2D) is associated with obesity. There is increasing evidence that T2D is associated with tumors [1] and cancers of the pancreas [2], prostate, breast, colon, endometrium, and liver [3]. T2D genes, such as *HNF-1 beta *and *JAZF1*, have been associated with prostate cancer [4-6]. Thus, T2D candidate genes may not only be obesity predisposing genes, but also tumor/cancer risk genes. CHOP mediates apoptosis and regulates mitochondrial gene expression, thus it may be implicated in beta cell inability to replicate as well as in insulin secretion defects. Following up on a linkage signal in the *CHOP *region of chromosome 12q13.1 in Italian T2D families, we have previously shown that *CHOP *5'UTR-c.279T>C and +nt30C>T haplotype variants are associated with early-onset T2D under a recessive and additive model [7]. In addition, CHOP inhibits adipogenesis [8], thus *CHOP *gene variants may contribute to insulin resistance [9,10] and/or obesity [11]. Since CHOP is regulating programmed cell death in response to stress stimuli [12], it is implicated in tumor/cancer development.

CHOP is involved in the pathogenesis of myxoid liposarcoma, a rare human tumor in which a reciprocal chromosomal translocation creates a fusion protein consisting of CHOP and TLS, a potent oncoprotein [13]. Other tumor-specific fusion genes, such as *EWS-CHOP *and *TLS/FUS-CHOP*, have been detected in solid tumors [14] and liposarcomas [15-17]. Another rearrangement of the *CHOP *gene has been reported in myxoid liposarcoma [18].

Our aim was to find whether there is any association of the *CHOP *5'UTR-c.279T>C and +nt30C>T genotypes and corresponding haplotypes (the latter contributing to T2D) [7] with body mass index (BMI) ≥ 25 (hereby defined as overweight condition/pre-obesity) and/or with tumors/cancer in an Italian dataset.

## Methods

We recruited from the general population in Italy 45 subjects with BMI ≥ 25 and 44 control subjects with BMI < 25 and 54 subjects with at least one cancer or at least one tumor and 43 control subjects with no history of tumor or cancer. We obtained the written informed consent from each subject and the approval from the Institutional Review Board accordingly to Helsinki Declaration guidelines. DNA samples were directly sequenced by PCR and an automated fluorescence sequencer with specific primers for the *CHOP *5'UTR-c.279T>C and +nt30C>T genotypes.

We calculated via 70% power and type 1 error probability of 0.05, detectable odds ratios for genotype association tests in our two datasets, using the prevalence of 31.3% for overweight condition [19] and the prevalence of 2.7% for tumors/cancer in the Italian population [20].

Via Chi-Square test statistics, we tested the alleles for departure from Hardy-Weinberg equilibrium (HWE) in our two datasets in cases and control subject groups, separately.

Via the Mantel-Haenszel algorithm, we tested the *CHOP *5'UTR-c.279T>C and +nt30C>T genotypes for association with BMI ≥ 25 and with tumors/cancer.

In addition, we performed model free and parametric haplotype associations tests (dominant, recessive and additive models) for BMI ≥ 25 and for tumors/cancer, independently (EHPLUS software) [21].

## Results

Risk odds ratios of 0.248/2.943 for genotypes association tests were detectable in the pre-obesity dataset. Risk odds ratios of 8.210 for genotype association tests were detectable in the tumors/cancer dataset.

All alleles tested in each group of the two datasets of BMI ≥ 25 and of tumors/cancer were not in departure from HWE.

We did not identify in our dataset any significant and valid association of the *CHOP *5'UTR-c.279T>C and +nt30C>T genotype variants with BMI ≥ 25 (Table 1) as well as with tumors/cancer patients (Table 2).

**Table 1:** *CHOP *5'UTR-c.279T>C and +nt30C>T genotype association with overweight condition (BMI ≥ 25).

**Genotype**	**45 Cases**		**44 Control Subjects**		**χ^2^**	**2-t P**	**OR**	**95% C.I**.
**5'UTRc.279T>C**	+	-	+	-				

TT	31	14	26	18	0.92	0.33	1.53	0.59–4.02

CT	13	32	18	26	1.41	0.23	0.59	0.22–1.55

CC	1	44	0	44	0.98	0.32	8	0.06–8

**+nt30C>T**								

TT	0	45	0	44			NA	

CT	13	32	17	27	0.94	0.33	0.65	0.24–1.71

CC	32	13	27	17	0.94	0.33	1.55	0.58–4.13

X^2 ^= Chi-Square, 2-t P = 2-tailed p-value, OR = odds ratio,

C.I. = confidence interval

**Table 2:** *CHOP *5'UTR-c.279T>C and +nt30C>T genotype association with tumors/cancer.

**Genotype**	**54 Cases**		**43 Control Subjects**		**χ^2^**	**2-t P**	**OR**	**95% C.I**.
**5'UTRc.279T>C**	+	-	+	-				

TT	35	19	27	16	0.04	0.83	1.09	0.44–2.73

CT	17	37	14	29	0.01	0.91	0.95	0.37–2.45

CC	2	52	2	41	0.05	0.81	0.79	0.08–8.28

**+nt30C>T**								

TT	2	52	1	42	0.15	0.69	1.62	0.11–46.73

CT	17	37	16	27	0.35	0.55	0.78	0.31–1.96

CC	35	19	26	17	0.19	0.66	1.20	0.48–2.99

X^2 ^= Chi-Square, 2-t P = 2-tailed p-value, OR = odds ratio,

C.I. = confidence interval

The parametric and non-parametric *CHOP *5'UTR-c.279T>C and +nt30C>T haplotype association tests with BMI ≥ 25 as well as with tumors/cancer were also not significant (data not shown).

## Discussion

*CHOP *gene encodes a C/EBP (CCAAT/enhancer binding protein family)-homologous nuclear protein that acts as dominant-negative inhibitor of gene transcription through dimerization with C/EBP [22]. *CHOP *is implicated in programmed cell death [12]. Several studies reported *CHOP *gene rearrangement and/or fusion with other genes (such as *EWS-CHOP *and *TLS/FUS-CHOP*) in tumors/cancer [13,18].

Cellular and endoplasmic reticulum (ER) stress, occurring in response to toxic and metabolic insult, is a powerful inducer of CHOP [12]. ER stress down-regulates insulin receptor signaling and triggers insulin resistance [9]. Furthermore, insulin increases *CHOP *expression in adipocyte cells [23], and CHOP inhibits adipocyte differentiation [8]. Thus, CHOP deficiency may contribute to obesity [11].

Glucotoxicity induces cellular stress [24], which activates CHOP [12]. Thus, hyperglycemia may cause CHOP-mediated beta-cell apoptosis and may contribute to T2D. Interestingly, *CHOP *5'UTR-c.279T>C and +nt30C>T haplotype variants are significantly associated with early-onset T2D under a recessive and additive model [7]. For all the above reasons, *CHOP *is not only a T2D gene, but it is also an obesity candidate gene as well as a gene potentially predisposing to tumors and/or cancer. Other T2D genes, such as *HNF-1 beta *and *JAZF1*, have already been associated with prostate cancer [4-6]. Of note, while the prostate cancer risk *HNF-1 beta *variant decreases the risk of T2D [4], variants of *JAZF1 *gene are associated with both increased risk for T2D and for prostate cancer [5,6].

However, no study has up to date investigated the susceptibility role of *CHOP *common variants in pre-obese and tumor/cancer patients. This is the first association study focusing on *CHOP *gene variants in human genomic DNA samples of overweight subjects and tumor/cancer cases.

In our study, we did not identify any association between *CHOP *5'UTR-c.279T>C and +nt30C>T genotype and haplotype variants with pre-obesity and with tumors/cancer. If the *CHOP *gene variants tested were to contribute to overweight condition and/or tumors/cancer with a modest size effect, our datasets are too small to detect such effects. However, we could at least exclude in the current study a *CHOP *5'UTR-c.279T>C and +nt30C>T variant risk effect of about 3 for pre-obesity and of about 8 for tumors/cancer.

## Conclusion

In summary, we conclude that *CHOP *5'UTR-c.279T>C and +nt30C>T variants, both at genotype and at haplotype level, are not contributing to the overweight condition and tumors/cancer in our dataset. However, other studies are warranted to further exclude the role of any other *CHOP *variants in obesity and in tumor/cancer predisposition. Since obesity is a preventable associated factor in several tumors/cancer [25] and in other co-morbidities [26], and, since tumors and cancer may be prevented and/or diagnosed at an earlier stage, genetic studies to identity overweight risk predisposition as well as tumors/cancer risk susceptibility should be further performed to guide disease prediction and prevention.

## Competing interests

The authors declare that they have no competing interests.

## Authors' contributions

CG participated in study design, DNA amplification, sequence reading, project coordination and manuscript drafting and revising. RM carried out the statistical analysis, reference collection, and manuscript drafting. NP and LP executed PCR set up, DNA amplification and sequence reading.

All authors have read and approved the manuscript.
